# Understanding adolescent substance abuse in a Northern Cape township: Educators’ experiences and perceptions

**DOI:** 10.4102/hsag.v31i0.3082

**Published:** 2026-01-13

**Authors:** Keikeditse E. Mohomane, Lizeka Napoles, Sanele Ngcobo, Nothando A.N. Mbatha

**Affiliations:** 1 School of Health Systems and Public Health, Faculty of Health Sciences, University of Pretoria, Pretoria, South Africa; 2 Department of Family Medicine, Faculty of Health Sciences, University of Pretoria, Pretoria, South Africa

**Keywords:** adolescent, learners, substance abuse, educator perceptions, educator experiences, life orientation curriculum

## Abstract

**Background:**

Learner substance abuse is a global public health concern contributing to preventable injuries, poor academic performance, and increased dropout rates. Addressing it in schools is crucial for improving both educational and health outcomes.

**Aim:**

This study explores high school educators’ perceptions and experiences of learner substance abuse.

**Setting:**

The study was conducted in six high schools in Galeshewe Township, Northern Cape province.

**Methods:**

A qualitative, exploratory and descriptive approach was employed. Sixteen Life Orientation (LO) educators were purposively sampled for in-depth interviews. Data were collected using a semi-structured guide until saturation. Trustworthiness was ensured through credibility, dependability, transferability, and confirmability. Thematic analysis followed Braun and Clarke’s six-step process, with transcription via Microsoft Word and Excel.

**Results:**

Four themes emerged: factors driving learner substance abuse, educational disruptions, educator training and support, and intervention strategies. Educators reported substance abuse as common both in school and off-premises especially among older boys. They also observed an increasing trend among younger learners and girls. It was linked to disruptive behaviour affecting teaching and learning. Educators felt unprepared to manage these issues and criticised the LO curriculum’s limitations.

**Conclusion:**

Educators emphasised the need for specialised training programmes to equip them with skills to manage learners involved in substance abuse. They also called for greater support from leadership in the Department of Education.

**Contribution:**

The study’s findings highlight the challenges of learner substance abuse in the Northern Cape and provide context-specific interventions that can be used to inform policy that addresses substance abuse in schools.

## Introduction

The abuse of substances among learners presents significant challenges to individuals, educational institutions and society as a whole (Mohale & Mokwena 2020; Mokwena & Huma 2014; Shuro & Waggie 2024). According to the American Psychiatric Association, substance use disorder (SUD) is a chronic, relapsing condition characterised by the compulsive use of a substance despite harmful consequences (American Psychiatric Association 2013). It involves cognitive, behavioural and physiological symptoms indicating loss of control over substance abuse (American Psychiatric Association 2013). Globally, the abuse of alcohol, tobacco and illicit substances (such as cannabis, amphetamines, cocaine and opioids) among learners presents a significant public health concern (Patel et al. 2016).

In 2023, the World Health Organization (WHO) reported that approximately 25% of learners aged 15 to 19 years globally had consumed alcohol, highlighting the urgency of addressing this emerging global health crisis (WHO 2024). An increasing number of learners are seeking help for substance abuse, but available financial resources are inadequate to meet the growing demand for prevention and treatment in high-prevalence areas (WHO 2024). The average age for South African learners to start using substances is now 12 years, reflecting a decline over time, likely because of increased substance exposure and availability in communities (Dada, Burnhams & Erasmus 2019). Early initiation increases the likelihood of developing addiction, leading to learners having difficulties in school and their social lives and other detrimental effects on their overall health (Fahimi et al. 2015; Walton, Avenant & Van Schalkwyk 2016).

Substance abuse has severe consequences, including accidental injuries, suicidal behaviours, depression, personality disorders, unplanned sexual encounters, increased rates of sexually transmitted infections and poverty (Mohale & Mokwena 2020; Tshitangano & Tosin 2016). These issues contribute to higher school dropout rates, poor academic performance, and increased school violence, affecting young people into adulthood (Khoza & Shilubane 2021; Mkhize & Shembe 2022). Socio-economic factors significantly influence learners’ substance abuse, with poverty driving learners to abuse substances as a coping mechanism (Morojele & Ramsoomar 2016). Peer pressure, a lack of parental supervision and limited recreational facilities further exacerbate the issue (Tshitangano & Tosin 2016). Rural schools, already disadvantaged, struggle to address substance abuse because of resource limitations (Shuro & Waggie 2024). Additionally, the normalisation of substances such as marijuana increases prevalence, while easy access to cannabis and methamphetamines undermines prevention efforts (Mkhize & Shembe 2022; Mohale & Mokwena 2020). Substance abuse is a growing problem in Northern Cape schools (Shuro & Waggie 2024). The National Youth Risk Behaviour Survey reports that the province has the highest percentage of learners using alcohol on school property and a 9.2% cannabis use rate below age 13, both higher than in other provinces (Modise & Raga 2022). Cocaine and heroin use rates (10.4%) also exceed the national average of 6.7% (Modise & Raga 2022). These statistics highlight the urgency for targeted interventions.

In Galeshewe, Kimberley’s largest township, learners’ substance abuse remains a major public health and education concern despite national policy interventions. Learners under the influence struggle with concentration, motivation and future preparedness (Khoza & Shilubane 2021; Mokwena & Huma 2014). Substance abuse also contributes to behavioural issues such as aggression and defiance, making classroom management challenging (Mkhize & Shembe 2022; Modise & Raga 2023). While research has focused on the health effects of learners’ substance abuse, educators’ role in prevention remains underexplored. As first responders to substance abuse behaviours, their perceptions are vital for effective intervention strategies (Walton et al. 2016). However, their experiences in managing and mitigating this issue require further investigation. This study’s aim is to explore high school educators’ perceptions and experiences regarding learners’ substance abuse in selected secondary schools in Galeshewe, Northern Cape.

## Research methods and design

A qualitative, exploratory and descriptive research design was used to investigate the educators’ perceptions and experiences regarding learners’ substance abuse in Galeshewe Township. This approach allowed participants to thoroughly explore and express their perceptions and experiences, providing in-depth insights.

### Research setting

This study was conducted in six secondary schools in Galeshewe (the oldest township) in the Northern Cape, which were purposefully selected as the study sites. These schools were selected because they serve as settings in which educators consistently deal with and manage learner substance abuse. Galeshewe faces significant socio-economic challenges, including a high prevalence of foetal alcohol spectrum disorder (FASD), which affects approximately 11% of young people per 1000 and has a profound impact on the local youth (Modise & Raga 2023).

### Study population

The study’s population consisted of Life Orientation (LO) educators at high schools. The focus was on permanent educators who have a minimum of 2 years of experience teaching LO to high school learners in grades 8 to 12. Life Orientation educators were selected because of their pivotal role in delivering health education within the LO curriculum, promoting health awareness and fostering a supportive school environment, all of which are vital in enhancing the teaching and learning experience (Joubert 2023). Educators in practical training or internships, those who did not have a minimum of 2 years of LO teaching experience, and those not teaching LO were excluded from the study.

### Sampling

A non-probability purposive sampling method was employed to ensure the collection of relevant and in-depth data (Creswell & Clark 2017). A total of 16 LO educators participated in the study, comprising 9 women and 7 men. The sample size follows the qualitative research principles recommended for qualitative studies (Naderifar, Goli & Ghaljaie 2017).

### Data collection procedures

Data collection took place from 03 May 2024 to 14 May 2024 using a semi-structured interview guide developed using current literature and refined after a pilot study. A pilot study was conducted with two participants from a high school in Warrenton, Northern Cape province.

Telephonic communication was initiated with school principals to seek their permission to conduct the study in their respective schools and approval for educators’ participation in the study. Subsequently, a meeting was convened with each of the principals from the selected schools, who acted as gatekeepers. The researcher initiated the process by introducing herself and providing detailed information regarding the study to the participants. Educators provided written informed consent, including permission for audio recording, before data collection. The interviews were audio-recorded to ensure accurate data capturing. Interviews explored educators’ perceptions of substance use among learners, challenges, parental roles, community involvement, and intervention strategies. The flexible format allowed for in-depth discussion while maintaining consistency. Interviews, conducted in English on school premises, lasted 40 min to 60 min in a private setting to ensure confidentiality. Data collection continued until saturation was reached with the 13th participant; however, three more participants were interviewed to confirm that no newer themes emerged. Ethical protocols were strictly followed, ensuring anonymity and data security.

### Data analysis

Data analysis was conducted concurrently with data collection, allowing the researcher to engage with the raw data in real time. Upon completion of the interviews, all data were transcribed into a MS Word document and analysed using thematic analysis, following Braun and Clarke’s framework (Byrne 2022). This approach involved systematically identifying, coding and categorising recurring patterns in the qualitative data to develop meaningful themes. To ensure accuracy, the researcher repeatedly reviewed audio recordings and annotated transcripts, highlighting key text segments. The transcripts were then organised into an MS Excel spreadsheet, where similar codes were grouped to form themes. The thematic analysis followed six phases: familiarisation with the data, generating initial codes, identifying potential themes, reviewing themes, defining and naming themes and producing the final report. Themes were systematically clustered based on similarities and differences to ensure consistency and validity. The final phase involved compiling a structured report that accurately reflected participants’ insights and experiences, providing a coherent interpretation of the findings.

The trustworthiness of the study was ensured by adhering to four key principles outlined by Creswell & Creswell (2017). Credibility was maintained through audio-recorded interviews and prolonged engagement with participants, ensuring in-depth and accurate data collection (Johnson, Adkins & Chauvin 2020). Dependability was achieved by systematically documenting data collection procedures, including field notes and recordings, with supervisory review to reduce potential biases (Williams & Moser 2019). Transferability was enhanced by selecting educators with direct experience working with learners in Galeshewe Township, a socio-economically challenged area, allowing findings to apply to similar contexts. Finally, confirmability was ensured through systematic data analysis, using direct participant quotes to maintain authenticity and accurately reflect their perspectives (Korstjens & Moser 2017).

### Ethical considerations

Ethical clearance was obtained from the Faculty of Health Sciences Research Ethics Committee, University of Pretoria (Ref: 668/2023), with additional approval from the Northern Cape Department of Education (Ref: L2, 10.2, 4, 3) and school principals. Participants were fully informed about the study’s purpose, potential benefits and risks, and written informed consent was obtained, including permission for audio recording.

## Results

This study included a total of 16 participants, consisting of 7 men and 9 women, as outlined in Table 1. The participants’ ages ranged from 25 to over 60 years, demonstrating a diverse population. Their teaching experience in LO ranged from 2 to over 22 years, offering a broad range of classroom experiences and perceptions on substance use among learners.

**TABLE 1:** The demographic information of the participants involved in the study.

Item	Categories (Years)	Frequency
Gender	Female	9
	Male	7
Age range (years)	25–34	3
	35–44	5
	45–54	4
	55–60+	4
Experience range (years)	2–4	3
	5–9	6
	10+	7

The thematic analysis of high school educators’ perceptions and experiences regarding substance abuse among learners in Galeshewe Township revealed four key themes, summarised in Table 2. These themes are factors driving the increase in learners’ substance abuse, disruptions to education, educator skilling and support, and adopted strategies for managing substance abuse.

**TABLE 2:** Themes and sub-themes on educators’ perceptions and experiences about learners’ substance use.

Themes	Sub-themes	Key findings
1. Factors driving the increase in learners’ substance abuse	1.1. Shifting demographics of substance abuse	Boys were previously the predominant users, but an increasing trend is observed among girls. Substance abuse is now common in Grades 8 to 12.
	1.2. Influence of popular culture on substance abuse	Social media, music and TV glamorise substance use. Celebrities and influencers make marijuana and alcohol appear socially acceptable
	1.3. Societal acceptance of learners’ substance abuse	Peer pressure and curiosity drive experimentation. A lack of parental guidance, broken homes and child-headed households increase the likelihood of abuse.
	1.4. Accessibility of substances in the community	Learners easily access substances in neighbouring hostels. No strict age restrictions on purchasing substances, even when wearing a school uniform.
	1.5. Socio-economic influences on substance abuse	Poverty and unemployment push learners to abuse substances as a coping mechanism. Some students sell drugs to support their families. Community members exploit learners as drug distributors.
	1.6. A lack of parental and community engagement	Parental involvement is minimal, with some parents struggling with substance abuse themselves.
2. Disruptions to education	2.1. Poor academic outcomes	Substance abuse leads to classroom disruptions, skipping classes and declining performance. Educators feel frustrated as learners who abuse substances negatively impact pass rates.
3. Educator skills and support	3.1. Specialised training	Educators feel ill-equipped to handle aggressive learners abusing substances. Urgent need for workshops on substance use intervention and management.
4. Adopted strategies for managing substance abuse	4.1. Short-term interventions	Most educators remove affected learners from class to maintain order. Some schools follow a structured disciplinary process using the Smart School Management System (SSMS). Community organisations such as ‘Wanya Tsotsi’ and ‘Operation Fiela’ assist in patrolling and bringing learners back to school. There is a lack of long-term interventions and proactive substance abuse policies.

### Theme 1: Factors driving the increase in learners’ substance use

In the study, the majority of participants reported encountering learners under the influence of substances daily, regardless of gender or age. *Marijuana* and *alcohol* were identified as the most commonly used substances, with learners increasingly exposed to them through *social media* and *television*.

#### Shifting demographics of substance use

Six participants (*P1, P2, P5, P8, P9 and P16*) observed that substance abuse was more prevalent among boys than girls:

‘We find that boys are the ones mostly using these substances.’ (P1, M, 7)

‘Statistically, males are more frequently identified as substance users. It is rare to identify girls.’ (P5, M, 7)

However, other participants (*P4, P6, P11, P13 and P14*) found a shifting trend, indicating that girls are now engaging in substance abuse as frequently as boys:

‘In previous years, substance abuse was predominantly a boys’ issue, but now, even girls are involved.’ (P4, F, 10)

‘There is a growing trend where girls are just as engaged as boys in using drugs and alcohol at school. There is no difference anymore.’ (P14, M, 4)

Additionally, substance abuse was reported across all grade levels, with a notable increase among younger learners:

‘I cannot say it’s only Grade 12 learners. All grades are affected.’ (P2, F, 30)

‘Grade 8 learners, around 13 years old, are now using substances. Previously, it was more common in Grades 11 and 12, but now the trend is shifting to younger learners.’ (P3, F, 13)

#### Influence of popular culture on substance use

Participants raised concerns about the role of *social media* and *television* in glamorising substance abuse:

‘Marijuana is popularised by celebrities, making it seem cool.’ (P8, F, 3)

‘Many of their role models use these substances, and it is glorified in music videos and media.’ (P14, M, 4)

While many participants attributed this trend to *social media influencers*, one participant highlighted the *lack of recreational activities* in *Galeshewe township* as another contributing factor:

‘There is a lack of alternative activities like sports, so drugs and alcohol become their only options.’ (P13, M, 4)

#### Societal acceptance of learners’ substance use

Participants observed that *societal dynamics*, such as *peer pressure* and *home environments*, significantly influence learners’ substance abuse. One educator remarked:

‘They might want to experience the feeling or fit in, driven by stories they hear or peer pressure.’ (P8, F, 3)

Another participant highlighted the role of *curiosity* and *environmental factors*:

‘It is curiosity, experimentation, and peer pressure. The situation and background also play a role.’ (P5, M, 7)

Educators emphasised that substance abuse is not solely an individual choice but often results from broader *societal pressures*, including a *lack of parental support, broken homes*, and *peer influence*:

‘Peer pressure is a significant contributing factor. Twelve educators pointed out that learners often succumb to peer influence to fit into certain groups, which impairs their ability to make independent decisions and frequently leads to substance experimentation.’ (P8, F, 3)

In addition, participants highlighted the role of *home environments*, particularly in *child-headed households*, in making learners more vulnerable to substance use. One educator opined:

‘Some learners come from homes where parents are constantly fighting.’ (P3, F, 13)

This often leads learners to turn to substances as a *coping mechanism* for *emotional distress*:

‘When you speak to these children one-on-one, you can see that the problem often starts at home or is influenced by what is happening in the community.’ (P9, M, 36)

#### Accessibility of substances in the community

Participants reported that substances were *easily accessible* in their community, particularly in nearby hostels:

‘Our school is near hostels and a notorious area, so these substances are easily accessible to learners.’ (P14, M, 4)

Furthermore, participants noticed that there were no effective restrictions preventing learners from purchasing substances:

‘I think it is a matter of accessibility. These substances are widely available. There is no age restriction preventing learners in uniform from purchasing them.’ (P6, F, 9)

#### Socio-economic influences on substance use

Participants highlighted that learners from *lower socio-economic backgrounds* were more likely to abuse substances as a *coping mechanism* because of *familial challenges*, such as *parental unemployment*:

‘Learners from poor backgrounds use substances to escape the harsh reality at home.’ (P5, M, 7)

‘Some learners also engage in selling substances because they lack a source of income at home.’ (P6, F, 9)

One educator expressed concern that *community members exploit vulnerable learners* by recruiting them to sell substances:

‘There is a local seller, a Rasta man, who supplies students with weed to sell to their peers.’ (P10, F, 2)

‘We have learners selling drugs on school premises. They might be working for someone in the community, possibly a drug lord.’ (P13, M, 4)

#### A lack of parental and community engagement

Participants highlighted the *lack of parental and community involvement* in supporting learners struggling with substance abuse. Many educators felt overwhelmed by the responsibility:

‘The school tries to create a relationship with parents and the community, but parents don’t want to be involved. They do not support us at all.’ (P10, F, 2)

‘There’s little collaboration. Parents often come to school under the influence of alcohol, which makes it hard to address the problem.’ (P8, F, 3)

Many participants expressed frustration over the lack of *community support* in addressing substance abuse among learners:

‘The community lacks involvement, as these concerns occur near the school premises.’ (P5, M, 7)

‘The community doesn’t seem to care about the school.’ (P9, M, 36)

### Theme 2: Disruptions to education

In this theme, participants expressed their frustrations regarding the disruptive behaviours that manifest when learners are under the influence of substances, affecting the classroom environment and disturbing teaching and learning.

#### Poor academic outcomes

Participants reported that substance use disrupts the classroom environment through behaviours, such as *late coming, making jokes and skipping lessons*. Educators emphasised that these behaviours negatively impact other learners’ ability to perform optimally:

‘Learners under the influence disturb others, skip classes, and require repeated instruction.’ (P5, M, 7)

‘It affects the teaching and learning process because when giving a lesson in class and then you have this one and others under the influence, you have to pause and address that matter.’ (P5, M, 7)

Of the educators who participated in the study, 63% expressed dissatisfaction with the poor academic performance of learners using substances, as their low grades impacted the educators’ pass rates, which are assessed on. Some participants observed:

‘Most of the learners who are smoking are underperforming. They underperform because they are never at school, always bunking to smoke. They use substances to fit in – if they can’t be achievers academically, they want to be seen as the cool or bad guys.’ (P8, F, 3)

‘Substance use particularly affects those with potential, making it harder for them to perform academically. Those who already struggle academically only make things worse.’ (P14, M, 4)

‘The passing rate goes down. When a significant portion of learners is using substances, some drop out, and others become pregnant, all of which negatively impact our results.’ (P11, M, 12)

### Theme 3: Educator skills and support

#### Specialised training

Participants emphasised the need for further training to equip educators with the skills to manage learners abusing substances. Educators acknowledged an *urgent need* for specialised workshops focusing on *substance abuse prevention and intervention*:

‘Yes, we need more training. Some teachers do not know how to handle these learners, especially when they become aggressive.’ (P7, F, 30)

Participants also stressed that training should extend beyond educators to include *parents and the broader school community*:

‘It is not only about training educators. Parents also need training because they play a crucial role. Both educators and parents need training.’ (P14, M, 4)

### Theme 4: Adopted strategies for managing substance abuse

To address substance abuse among learners, educators employ various strategies, including *removing learners from class* and *reporting incidents to the principal*. These approaches highlight the urgent need for *long-term interventions* to create a *conducive teaching and learning environment*.

#### Short-term interventions used to manage substance abuse

Educators held differing views on how to handle learners under the influence of substances. The most commonly reported approach was *removing affected learners from the classroom* to minimise disruptions. Participants indicated:

‘I remove them from the classroom to maintain peace, but this is not a long-term solution.’ (P10, F, 2)

‘The easiest way sometimes is for the teacher to get them out of the class so that I can continue teaching the others.’ (P9, M, 36)

Other participants emphasised adherence to the *substance abuse policy*:

‘The standard procedure is to report the learner to the office, where the case is registered in their profile on the Smart School Management System [*SSMS*]. The disciplinary committee, including representatives from the learners [*RCL*], parents [*SGB*], and school management [*SMT*], then takes over.’ (P14, M, 4)

‘The child will still be allowed to attend school after the first positive test. The policy also dictates that after 30 days, we shall retest the learner.’ (P3, F, 13)

One participant suggested that continuous collaboration between the community and schools – particularly with community groups, such as ‘Wanya Tsotsi’ and ‘Operation Fiela’ – could be beneficial in *monitoring the behaviour of substance-abusing learners and encouraging them to return to school*:

‘There’s a lady here just opposite the school. She went to the extent of patrolling the nearby tuck shops early in the morning. There are Non-Governmental Organizations [*NGOs*] like “Operation Fiela” [*Operation Sweep*] and “Wanya Tsotsi” [*meaning “the criminals will defecate”*], who also help us in patrolling. Especially when they see a child wearing a school uniform, they make it their mission to bring the child back to school. So yes, the relationship is very effective.’ (P3, F, 13)

The study’s findings revealed a *lack of tailored interventions* to support educators by equipping them with *skills and knowledge* regarding substance use policies in educational settings.

## Discussion

This study explored high school educators’ perceptions and experiences regarding learner substance abuse in Galeshewe Township, Kimberley, Northern Cape province, South Africa. The study’s findings revealed that educators frequently encountered learners under the influence of substances both on and off school premises, mostly during intervals. Participants perceived marijuana and methamphetamine (‘tik’) as commonly used substances, while alcohol consumption was found to be more prevalent during school events. These findings align with previous research, which indicated that these substances remain common among learners (Dada et al. 2019; Nxumalo & Nel 2024; Peltzer & Phaswana-Mafuya 2018).

According to the findings, the number of girls abusing substances has been on the rise; however, substances are still predominantly used by male learners. This aligns with the findings of Tshitangano and Tosin (2016) in the Vhembedzi district of Limpopo, which indicated that male learners were more likely to use and abuse substances compared to girls. Educators noticed that male learners often struggled to express their challenges, such as family conflicts, which may contribute to their substance abuse. In contrast, educators perceived female learners as more likely to communicate their challenges.

The study also highlighted a significant trend showing that girls are now abusing substances at rates comparable to boys. This development is consistent with the report by Mohale and Mokwena (2020), which noted an increase in substance abuse among female learners. The participants in the study also initially held the perception that substance abuse was predominantly an issue among older learners in the 11th and 12th grades. However, they have observed a troubling trend of learners, some as young as 13, starting to engage in substance abuse. This trend is consistent with the research conducted by Dada et al. (2019) and Tshitangano and Tosin (2016), which indicated that the average age for the initiation of substance use among learners is around 13 years or even younger.

Popular culture, especially through social media and television, significantly influences the behaviour of learners abusing substances. These media often portray substance abuse in a way that appears fashionable or normalised, which may lead learners to perceive such behaviours as socially acceptable. This effect is intensified in the absence of parental guidance and supervision, particularly in terms of directing learners and limiting their access to specific social media platforms (Rayn, Roman & Okwany 2015). In addition, a lack of adequate recreational facilities further intensifies these issues, limiting the availability of healthy leisure activities. Consequently, learners may resort to substance abuse as a means of coping with the lack of alternatives (Tshitangano & Tosin 2016).

The study also highlighted the significant impact of peer pressure on learners, who often feel pressured to conform to the behaviours of their peers to feel socially acceptable. This finding is consistent with previous research that demonstrates a strong relationship between peer influence and substance use among learners in school settings (Idowu et al. 2018; Khoza & Shilubane 2021). Furthermore, family conflict was identified as a significant factor leading to substance abuse, with learners resorting to these substances as a coping mechanism. This observation is supported by the research of Nzama and Ajani (2021), which found that familial challenges, neglect, and a lack of attention significantly contribute to substance abuse among learners.

The availability of substances within the community was also highlighted as a significant concern. Participants noticed that substances, such as marijuana and other illegal substances, are readily accessible, especially in proximity to hostels and recognised hotspots for illegal substance activities. This observation corroborates the findings of Tshitangano and Tosin (2016), who emphasised accessibility as a crucial determinant of substance abuse among learners in rural settings. Furthermore, this is consistent with the research by Mohale and Mokwena (2020), which indicated that areas with low socio-economic status tend to have a higher concentration of shebeens and unlicensed alcohol vendors, thereby enhancing the availability of illicit substances to learners.

This study also highlighted that learners from economically disadvantaged backgrounds are more susceptible to substance abuse, influenced by financial difficulties and family instability. Educators emphasise that parental unemployment and community exploitation, especially in the recruitment of learners for substance sales, intensify this issue. This aligns with Manu, Douglas and Ayanore (2020) and Peltzer and Phaswana-Mafuya (2018), who found that economic hardship and cannabis legalisation contributed to increased substance abuse.

Participants highlighted the negative impact of substance abuse on academic performance, particularly regarding concentration and comprehension. Educators noticed that learners abusing substances often struggle to engage in lessons, aligning with Mokwena and Huma (2014), who found that these learners have difficulty focusing and understanding material. The addictive nature of substances further complicates cognitive development and mental health of learners (Mokwena & Setshego 2021). Such learners often disengage, neglect homework and disrupt class, hindering the learning process. This aligns with research by Shuro and Waggie (2024) and Mohale and Mokwena (2020), which collectively highlight decline in academic performance and participation among substance users.

The significance of specialised training for educators in effectively addressing substance abuse in the classroom has been emphasised. Such training, which prioritises prevention and intervention, is deemed essential for equipping educators to detect warning signs, implement effective strategies and provide targeted support. This necessity for professional development corresponds with wider calls for a cooperative strategy, where schools, families and communities collaborate to address learners’ substance abuse. The Policy Framework for the Management of Drug Abuse in Schools highlights that the responsibility for this issue cannot rest solely on educators (Tshitangano & Tosin 2016). Tshitangano and Tosin (2016) further assert that parental involvement is crucial in aiding learners affected by substance abuse, thus underscoring the need for a collective response to this challenge.

The research highlighted a significant lack of tailored interventions to equip educators with essential skills and knowledge about substance abuse policies in schools. This aligns with Manu et al. (2020), who found that educators often have limited understanding of South African legislation on substance abuse. Educators in resource-constrained communities, such as Galeshewe, face complex challenges including poverty, unemployment and limited parental supervision, which increase learners’ susceptibility to substance abuse. The lack of recreational programmes further worsens this vulnerability. Many educators often feel unequipped to manage the behavioural and emotional impact of substance abuse, revealing a gap between policy design and practical implementation. By aligning strategies with community and learner contexts, these interventions can address the gap between policy and practice (Ngubane 2021).

### Strengths and limitations

This research provided valuable insights into educators’ perceptions and experiences of learners’ substance abuse, improving understanding of their role in prevention initiatives. Purposive sampling ensured participants had relevant knowledge, yielding rich data. By focusing on educators in low-income schools, the study highlights unique challenges and opportunities for targeted interventions.

However, the study also had limitations. Participant bias may have influenced the findings, as responses are based on individual experiences and perspectives. While the study provides meaningful insights, its transferability to other educational settings is limited because of its focus on specific schools in a low-income context. Additionally, the qualitative nature of the research means that findings are shaped by personal narratives, which potentially limits their generalisability. Furthermore, because this study was conducted during school hours, some educators who met the inclusion criteria were unable to participate because of work commitments.

### Recommendations

The following recommendations are suggested to help mitigate the impact of substance abuse on learners and support educators and learners in the school environment.

Firstly, schools should consider implementing targeted intervention programmes that focus on the negative effects of substance use and preventative strategies. These programmes should be tailored to the specific needs of the learners and involve training for educators, parents and the wider community on how to engage and support learners affected by substance abuse.

Secondly, schools should employ professionals, such as nurses, social workers and security personnel to provide immediate support for both learners and educators. The presence of these professionals would ensure that timely intervention is available for those experiencing substance use-related challenges or other social challenges.

The study also highlighted the importance of proper referrals and rehabilitation support for learners affected by substance abuse. According to the Department of Education (2008) and Ngubane (2021), the *South African Schools Act* empowers principals or designated school officials to conduct drug testing on learners, following strict procedures to ensure fairness and protect learners’ rights. Schools need to work with health professionals and social services to provide support and intervention for affected learners. Affected learners should have access to rehabilitation centres and professional support tailored to their needs.

Lastly, schools should create a counselling programme where learners can seek confidential support from trained professionals. These programmes would provide a safe space for learners to discuss their challenges and receive appropriate guidance. To ensure that substance abuse is being effectively managed, schools should assess the effectiveness of their existing substance use policies using standardised evaluation tools. School-based strategies for substance use prevention must be based on the principles of intersectoral collaboration integrated into National Health Policies (Shuro & Waggie 2021). This would promote ongoing improvements and ensure that the intervention remains significant and effective. National Drug Master Plan (NDMP), which outlines the intersectoral response to substance abuse prevention in South Africa, should be used to guide intervention strategies by schools (Ngubane 2021).

## Conclusion

The study’s findings highlight the challenges faced by educators in addressing learner substance abuse, which contributes to disruptive behaviour and negatively impacts the teaching and learning environment. The lack of training and inadequacies within the LO curriculum further exacerbate the issue. The study highlights the need for comprehensive interventions, including enhanced educator support and specialised training, to empower educators to effectively address substance abuse, promoting a safer and more conducive learning environment.

## References

[CIT0001] American Psychiatric Association, 2013, *Diagnostic and statistical manual of mental disorders: DSM-5*, 5th edn., American Psychiatric Association, Washington, DC.

[CIT0002] Byrne, D., 2022, ‘A worked example of Braun and Clarke’s approach to reflexive thematic analysis’, *Quality & Quantity* 56(3), 1391–1412. 10.1007/s11135-021-01182-y

[CIT0003] Department of Education, 2008, *Guidelines for random search and seizure and drug testing at schools. Annexure B, Government Gazette*, No. 31417, viewed 1 June 2025, from https://www.westerncape.gov.za/education/files/wcg-blob-files?file=2025-01/23_09_b_annb.pdf&type=file.

[CIT0004] Creswell, J.W. & Clark, V.L.P., 2017, *Designing and conducting mixed methods research*, 3rd edn., Sage, Thousand Oaks, CA.

[CIT0005] Creswell, J.W. & Creswell, J.D., 2017, *Research design: Qualitative, quantitative, and mixed methods approaches*, 5th edn., Sage, Thousand Oaks, CA.

[CIT0006] Dada, S., Burnhams, N.H. & Erasmus, J., 2019, *South African community epidemiology network on drug use (SACENDU): Monitoring alcohol, tobacco and other drug abuse treatment admissions in South Africa. Phase 45*, South African Medical Research Council, Cape Town, viewed 03 February 2024, from https://www.samrc.ac.za/sites/default/files/attachments/2019-10-16/SACENDUFullReportPhase45.pdf.

[CIT0007] Fahimi, J., Aurrecoechea, A., Anderson, E., Herring, A. & Alter, H., 2015, ‘Substance abuse and mental health visits among adolescents presenting to US emergency departments’, *Pediatric Emergency Care* 31(5), 331–338. 10.1097/PEC.0000000000000421 25875990 PMC4417003

[CIT0008] Idowu, A., Aremu, A.O., Olumide, A. & Ogunlaja, A.O., 2018, ‘Substance abuse among students in selected secondary schools of an urban community of Oyo-state, South West Nigeria: Implication for policy action’, *African Health Sciences* 18(3), 776–785. 10.4314/ahs.v18i3.36 30603011 PMC6307013

[CIT0009] Johnson, J.L., Adkins, D. & Chauvin, S., 2020, ‘A review of the quality indicators of rigor in qualitative research’, *American Journal of Pharmaceutical Education* 84(1), 7120. 10.5688/ajpe7120 32292186 PMC7055404

[CIT0010] Joubert, C., 2023, ‘Life orientation teachers’ perspectives on a pastoral approach to the topic: “Development of the self in society”’, *South African Journal of Education* 43(2), 1–9. 10.15700/saje.v43n2a2227

[CIT0011] Khoza, A. & Shilubane, H.N., 2021, ‘Substance use and associated factors among in-school adolescents in South Africa’, *The Open Public Health Journal* 14(1), 435–440. 10.2174/1874944502114010435

[CIT0012] Korstjens, I. & Moser, A., 2017, ‘Series: Practical guidance to qualitative research. Part 4: Trustworthiness and publishing’, *European Journal of General Practice*, 24(1), 120–124, viewed 10 March 2024, from 10.1080/13814788.2017.1375092. 29202616 PMC8816392

[CIT0013] Manu, E., Douglas, M. & Ayanore, M.A., 2020, ‘Socio-ecological influences of adolescence marijuana use initiation: Qualitative evidence from two illicit marijuana-growing communities in South Africa’, *South African Journal of Psychiatry* 26(1), 1–11. 10.4102/sajpsychiatry.v26i0.1477 PMC7479363 32934841

[CIT0014] Mkhize, T.R. & Shembe, Z., 2022, ‘Intoxicated in the classroom: Teachers’ experiences of teaching drug addicted learners in KwaZulu-Natal township schools, South Africa’, *African Perspectives of Research in Teaching and Learning* 6(2), 29–43, viewed 24 March 2024, from https://www.researchgate.net/publication/364302515.

[CIT0015] Modise, J.M. & Raga, K., 2022, ‘Illicit use of Drugs among the Youth and Adults in the Frances Baard Region: Northern Cape, South Africa’, *International Journal of Innovative Science and Research Technology* 7(6), 677–686.

[CIT0016] Modise, J.M. & Raga, K., 2023, ‘Youth in the Northern Cape, South Africa, engage in high-risk behaviors such as drinking and using drugs’, *Recent Trends in Arts and Social Studies* 3, 71–81. 10.9734/bpi/rtass/v3/5793E

[CIT0017] Mohale, D. & Mokwena, K.E., 2020, ‘Substance use amongst high school learners in the south of Johannesburg: Is this the new norm?’, *South African Family Practice* 62(1), e1–e6. 10.4102/safp.v62i1.5122 PMC8378201 33314944

[CIT0018] Mokwena, K.E. & Huma, M., 2014, ‘Experiences of “nyaope” users in three provinces of South Africa: Substance abuse’, *African Journal for Physical Health Education, Recreation and Dance* 20(suppl. 1), 352–363, viewed 6 August 2024, from https://hdl.handle.net/10520/EJC162264.

[CIT0019] Mokwena, K.E. & Setshego, N.J., 2021, ‘Substance abuse among high school learners in a rural education district in the Free State province, South Africa’, *South African Family Practice* 63(1), e1–e6. 10.4102/safp.v63i1.5302 PMC8424709 34476961

[CIT0020] Morojele, N.K. & Ramsoomar, L., 2016, ‘Addressing adolescent alcohol use in South Africa: CME’, *South African Medical Journal* 106(6), 551–553. 10.7196/samj.2016.v106i6.10944

[CIT0021] Naderifar, M., Goli, H. & Ghaljaie, F., 2017, ‘Snowball sampling: A purposeful method of sampling in qualitative research’, *Strides in Development of Medical Education* 14(3), e67670. 10.5812/sdme.67670

[CIT0022] Ngubane, N.B., 2021, ‘Combating substance abuse amongst learners in South African schools’, Master’s dissertation, University of KwaZulu-Natal, Durban, viewed 6 January 2025, from https://researchspace.ukzn.ac.za/handle/10413/22240.

[CIT0023] Nxumalo, V.W. & Nel, Y.M., 2024, ‘Substance use patterns in an adolescent psychiatric unit in Johannesburg, South Africa’, *South African Journal of Psychiatry* 30, 2198. 10.4102/sajpsychiatry.v30i0.2198 PMC10839199 38322179

[CIT0024] Nzama, M.V. & Ajani, O.A., 2021, ‘Substance abuse among high school learners in South Africa: A case study of promoting factors’, *African Journal of Development Studies (formerly AFFRIKA Journal of Politics, Economics and Society)* 2021(si1), 219–242. https://doi.org/10.2634-3614

[CIT0025] Patel, V., Chisholm, D., Parikh, R., Charlson, F.J., Degenhardt, L., Dua, T. et al., 2016, ‘Addressing the burden of mental, neurological, and substance use disorders: Key messages from disease control priorities, 3rd edition’, *The Lancet* 387(10028), 1672–1685. 10.1016/S0140-6736(15)00390-6 26454360

[CIT0026] Peltzer, K. & Phaswana-Mafuya, N., 2018, ‘Drug use among youth and adults in a population-based survey in South Africa’, *South African Journal of Psychiatry* 24(1), 1–6. 10.4102/sajpsychiatry.v24i0.1139 PMC6138152 30263218

[CIT0027] Rayn, J., Roman, N.V. & Okwany, A., 2015, ‘The effects of parental monitoring and communication on adolescent substance use and risky sexual activity: A systematic review’, *The Open Family Studies Journal* 7(1), 12–27. 10.2174/1874922401507010012

[CIT0028] Shuro, L. & Waggie, F., 2021, ‘A vibrant reflection of the revised integrated school health policy with a lens on substance use’, *African Journal of Primary Health Care & Family Medicine* 13(1), a3082. 10.4102/phcfm.v13i1.3082 PMC8603133 34797121

[CIT0029] Shuro, L. & Waggie, F., 2024, ‘Trends in socio-demographic characteristics and substance use among high school learners in a selected district in Limpopo Province, South Africa’, *BMC Public Health* 24(1), 1407. 10.1186/s12889-024-18927-7 38802772 PMC11129414

[CIT0030] Tshitangano, T.G. & Tosin, O.H., 2016, ‘Substance use amongst secondary school students in a rural setting in South Africa: Prevalence and possible contributing factors’, *African Journal of Primary Health Care and Family Medicine* 8(2), e1–e6. 10.4102/phcfm.v8i2.934 PMC4845911 27380837

[CIT0031] Walton, K.L., Avenant, J. & Van Schalkwyk, I., 2016, ‘Educators’ experiences of their relationships with adolescents involved in drug use’, *South African Journal of Education* 36(3), 1–10. 10.15700/saje.v36n3a1188

[CIT0032] Williams, M. & Moser, T., 2019, ‘The art of coding and thematic exploration in qualitative research’, *International Management Review* 15(1), 45–55. https://doi.org/10.14286482

[CIT0033] World Health Organization. (2024). *Global status report on alcohol and health and treatment of substance use disorders*, World Health Organization, Geneva.

